# Turning NO_x_ Pollutants into Green Ammonia Fuel via COF Cavity Confinement

**DOI:** 10.3390/molecules31213938

**Published:** 2026-11-01

**Authors:** Marlene Bosquez, Roberto Salcedo

**Affiliations:** 1 Institute of Chemistry, ELTE Eötvös Loránd University, H-1117 Budapest, Hungary; 2 Hevesy György PhD School of Chemistry, ELTE Eötvös Loránd University, H-1117 Budapest, Hungary; 3 Instituto de Investigaciones en Materiales, Universidad Nacional Autónoma de México, Circuito Exterior S/N, C.U., Ciudad de Mexico 04510, Mexico

**Keywords:** NO_x_, sustainable ammonia, renewable energy, covalent organic framework, Haber–Bosch

## Abstract

The development of sustainable fuels and hydrogen carriers is essential for a carbon-neutral energy future. Ammonia (NH_3_) is a promising energy vector, yet its synthesis remains dependent on the energy-intensive Haber–Bosch process, while nitrogen oxides (NO_x_) persist as major atmospheric pollutants. In this work, spin-polarized density functional theory (DFT) calculations at the MPWB1K/def2SVP level are used to investigate the capture and reduction of NO and NO_2_ within a rigid covalent organic framework (M1). Nucleus-Independent Chemical Shift (NICS) analysis reveals a strongly aromatic shielding character (NICS = −10.8) that provides a unique electronic environment, while electrostatic potential (ESP) mapping illustrates local electron density. Confinement within the framework suppresses competing pathways that preferentially favor the pathway toward NH_3_ and H_2_O. Activation barriers are significantly reduced, driven by charge transfer that weakens N–O bonds. The rigid covalent organic framework remains structurally stable throughout the process. These findings demonstrate a viable strategy for converting atmospheric pollutants into valuable energy carriers under moderate thermal activation.

## 1. Introduction

Ammonia (NH_3_) is a vital pillar of modern industry, serving as an indispensable component in global agriculture, textiles, and pharmaceuticals [1]. Beyond these traditional applications, ammonia has recently attracted significant attention as a clean, sustainable green fuel and a high-capacity carrier for hydrogen energy storage [2,3,4,5]. Despite its potential, the global standard for ammonia production remains the classic Haber–Bosch process [6,7,8]. This conventional method requires extreme operational conditions, typically exceeding 400 °C and 100 atmospheres of pressure [8,9]. Furthermore, because the Haber–Bosch process relies heavily on fossil fuel feedstocks, such as methane, for hydrogen production [9,10,11,12], it is a massive driver of climate change, generating an annual CO_2_ emission level of 400 Mt [13,14,15]. Therefore, developing sustainable, low-energy alternatives for ammonia synthesis that bypass these limitations is a critical priority for decarbonization [5,7].

Simultaneously, the accumulation of atmospheric pollutants, such as nitrogen oxides (NO_x_), poses a severe environmental threat, leading to the chemical formation of nitric acid (HNO_3_) and the subsequent crisis of acid rain [13,14,15]. While conventional catalytic technologies successfully reduce automotive NO_x_ emissions into inert dinitrogen (N_2_) and oxygen (O_2_) [16], translating these systems efficiently to large-scale stationary industrial plants remains a challenge. Consequently, research has expanded into advanced remediation strategies, including photocatalytic and electrochemical cleanup [17,18,19]. While photocatalysis has proven excellent for environmental decontamination and nitrogen fixation [19], electrocatalysis offers a unique “circular” opportunity: converting reactive nitrogen species directly into valuable ammonia at room temperature and atmospheric pressure [2,3,17,18,20]. By utilizing sustainable reducing agents, such as renewable hydrogen derived from green water splitting [11,12], this strategy solves two ecological problems at once: mitigating hazardous air pollution while generating a valuable chemical feedstock [18].

**Figure 1:**
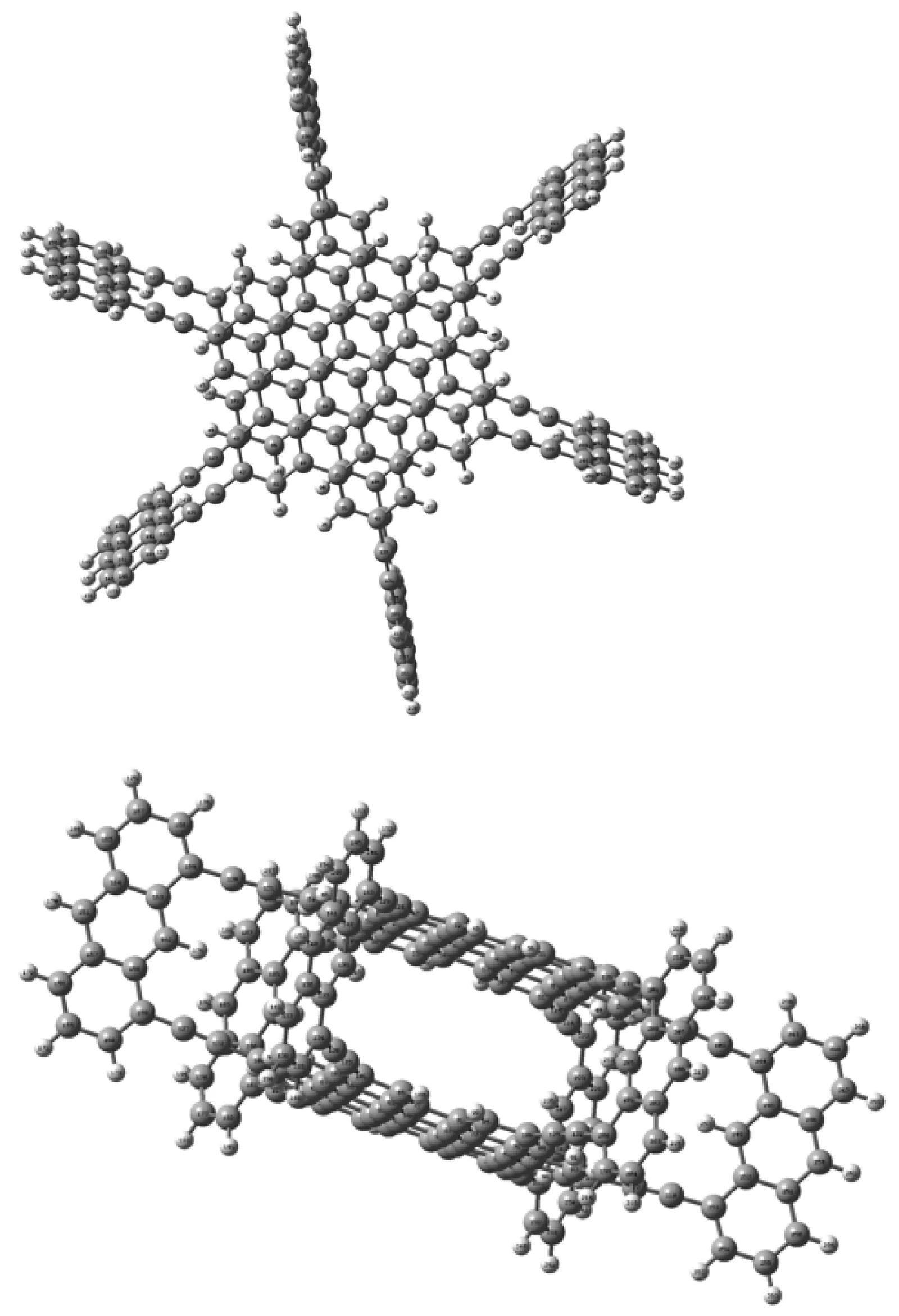
Structural representations of M1, a COF in which two polyaromatic decks are joint by anthracene units forming the catalytic hollow.

Covalent organic frameworks (COFs) [21,22,23] have emerged as highly promising materials to drive these sustainable transformations. COFs are crystalline, porous organic structures built from strong lattices of light elements, such as carbon, nitrogen, oxygen, and hydrogen. Because of their exceptionally large surface areas, low densities, and tunable pore sizes, COFs are ideal candidates for customized adsorption and catalytic applications [24,25]. In a previous study, our group introduced the theoretical design of a novel macrocyclic COF architecture, designated here as M1 (Figure 1) [21]. This unique species generates an electron-rich region shielded within a hollow chamber between two aromatic moieties. Our initial simulations demonstrated that this electron density could be successfully leveraged to capture and reduce carbon dioxide (CO_2_) into formic acid in the presence of hydrogen (H_2_) [21]. While the interactions of atmospheric NO_x_ with various frameworks have been studied theoretically, prior computational works have predominantly focused on molecular adsorption or photophysical reduction procedures [26,27]. To the best of our knowledge, a well-defined molecular framework acting as a localized reactor for the direct chemical reduction of gas-phase NO_x_ has not yet been fully explored.

From a technological perspective, using COFs for NO_x_ abatement relies on a heterogeneous continuous-flow fixed-bed configuration. The porous COF serves as a solid adsorbent bed that selectively captures and encapsulates gas-phase NO_x_ molecules from exhaust streams through strong host–guest interactions (π-stacking and electrostatic confinement). Co-feeding a stoichiometric stream of H_2_ gas (sourced via renewable water electrolysis) across the COF bed enables the localized, selective reduction of NO_x_ into green ammonia and water under thermal activation. While this catalytic process yields ammonia directly using H_2_ as a reducing agent, the ultimate designation of the produced NH_3_ as “green ammonia” is strictly contingent upon sourcing H_2_ from renewable water electrolysis rather than fossil-fuel-based steam methane reforming.

In this work, we use computational simulations to evaluate the catalytic reduction of open-shell NO and NO_2_ molecules trapped inside the molecular chamber of M1 using H_2_ as the reducing agent. Given the high concentration of electrons trapped within the hollow cavity of M1, we hypothesized that the framework could effectively stabilize and reduce these open-shell pollutants. Our results demonstrate that the M1 cage fundamentally alters the reaction pathway; instead of yielding atmospheric nitric acid [15], the process selectively converts the pollutants into ammonia and water. We present a detailed thermodynamic and kinetic analysis of this multi-step mechanism, establishing a theoretical roadmap for a promising, low-energy route to green ammonia synthesis from environmental waste.

## 2. Results and Discussion

### 2.1. Geometrical and Electronic Characterization of the M1

The covalent organic framework (COF) used in this study, denoted M1, corresponds to the structure previously reported by Rios and Salcedo [21]. This framework features two parallel polycyclic aromatic decks (coronene/circulene units) interconnected by six anthracene bridges, which enforce a rigid, eclipsed arrangement. The selection of anthracene linkers is motivated by their favorable performance in prior CO_2_ fixation studies [21]. The resulting architecture creates a confined, electron-rich cavity arising from the combined π-electron delocalization of both aromatic surfaces. Although this arrangement resembles a π–π stacking interaction, a key distinction lies in its structural rigidity: the bridging units prevent relative displacement of the decks, maintaining a fixed geometry and inhibiting framework collapse or distortion under reactive conditions. This persistent structural integrity suggests that M1 exhibits significant mechanical stability and potential recyclability, in agreement with related covalent organic frameworks and hybrid porous materials [28]. To model the catalytic cavity, a finite molecular cage representing the core building unit of the covalent organic framework (COF), designated as M1, was employed. While designated as M1 within the context of framework materials, all quantum mechanical calculations utilize this single, well-defined molecular cage to capture the localized host–guest interactions and confinement effects within the pore.

**Figure 2:**
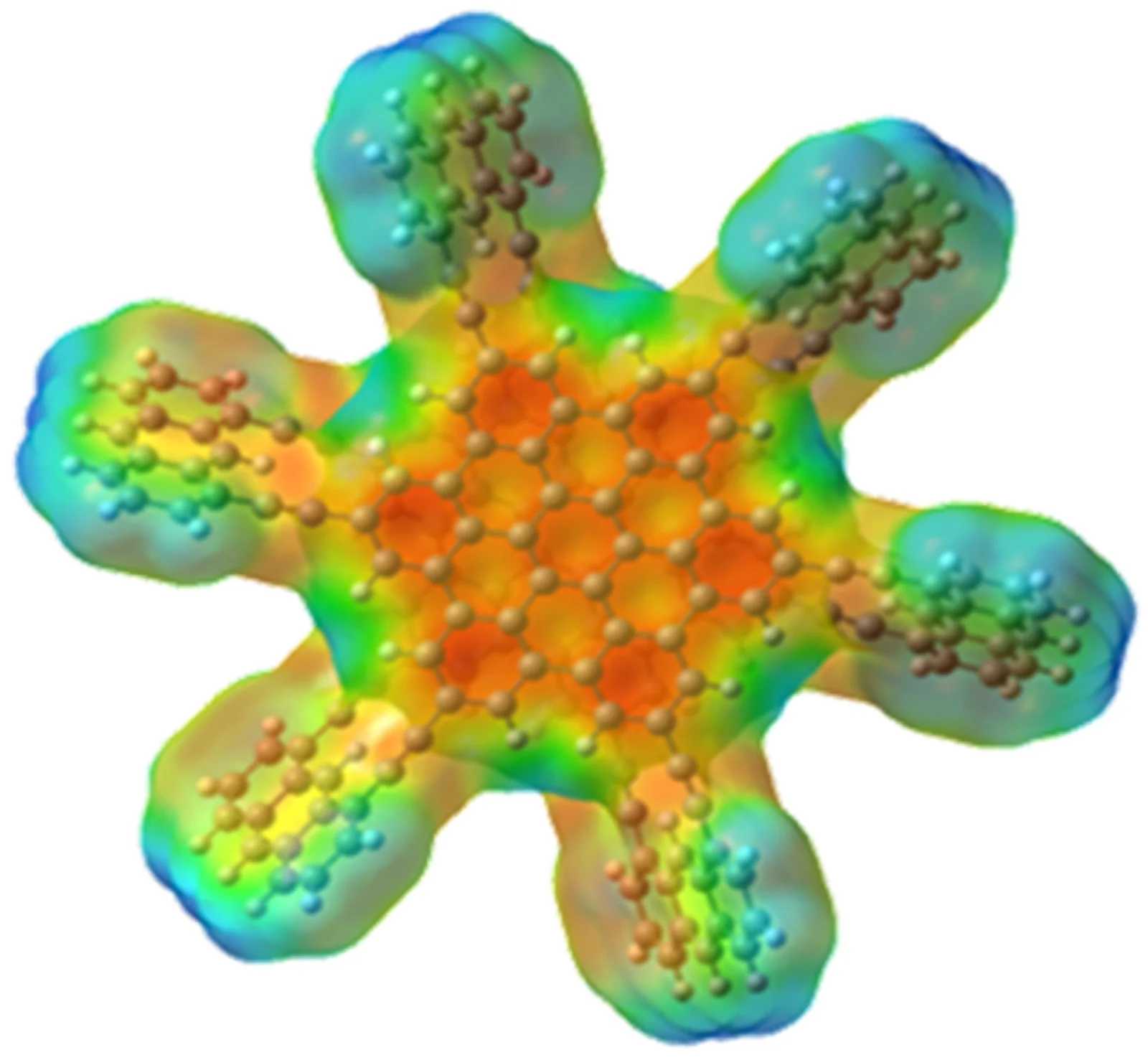
Electrostatic potential mapped on the electronic density for the selected catalytic chamber [21]. Red zones imply strong electron density, green neutral space whereas blue are positive charge density regions.

To quantitatively evaluate the catalytic environment inside the framework cavity, electrostatic potential (ESP) maps and NICS analyses were performed. The ESP map reveals a profound accumulation of electron density localized within the central hollow region bounded by the flanking aromatic decks [21] (Figure 2). This electron-rich environment is further characterized using NICS calculations [29], which yield values of −17.1 for the central zone of the deck, −13.7 for the edge regions, and −10.8 for the exact midpoint between the two parallel surfaces. These highly negative NICS values confirm a continuous, strongly aromatic character within the interior chamber. This localized electronic field serves as a specialized reaction site capable of interacting dynamically with encapsulated guest molecules, a phenomenon reminiscent of electronic accumulation and catalytic effects reported in advanced nanographene and cyclophane chemistry [30,31].

### 2.2. Thermodynamic Evaluation and Selective Reduction

Traditional non-confined NO_x_ reduction processes frequently yield a mixture of environmental by-products, including N_2_ and nitrous oxide (N_2_O), depending on temperature, active species, and phase boundaries [32,33]. However, when the reactive open-shell species are introduced into the confined chamber of M1, the simulations demonstrate complete selectivity toward a single pair of products: ammonia (NH_3_) and water (H_2_O). This behavior contrasts with conventional industrial Haber–Bosch pathways that demand extreme conditions to overcome high dissociation barriers [6,7,8].

The overall stoichiometric reactions modeled inside the M1 are defined as follows:

2NO_2_ + 7H_2_ → 2NH_3_ + 4H_2_O

2NO + 5H_2_ → 2NH_3_ + 2H_2_O

To validate the thermodynamic driving force of these transformations, the Gibbs free energy profiles were calculated for the unconstrained free reactions (FRs) and the corresponding reactions taking place inside the cage (IC), as summarized in Table 1. The frequency calculations indicate that encapsulation within the M1 cage systematically stabilizes the overall reaction thermodynamics compared with standard thermodynamic values (STVs), driven by the favorable host–guest non-covalent interactions within the cavity.

### 2.3. Kinetic Profiles and Confinement Effects

The computational M1 cage model represents the active pore environment within the heterogeneous COF matrix. In a catalytic reactor setup, NO_x_ species (NO and NO_2_) are captured from the continuous gas phase via non-covalent confinement inside the electron-rich M1 cavity. Simultaneously, co-fed H_2_ molecules diffuse into the occupied host–guest channels, bringing the reactants into close proximity (2.0 Å) to initiate step-wise hydrogenation without requiring noble metal centers.

**Figure 3:**
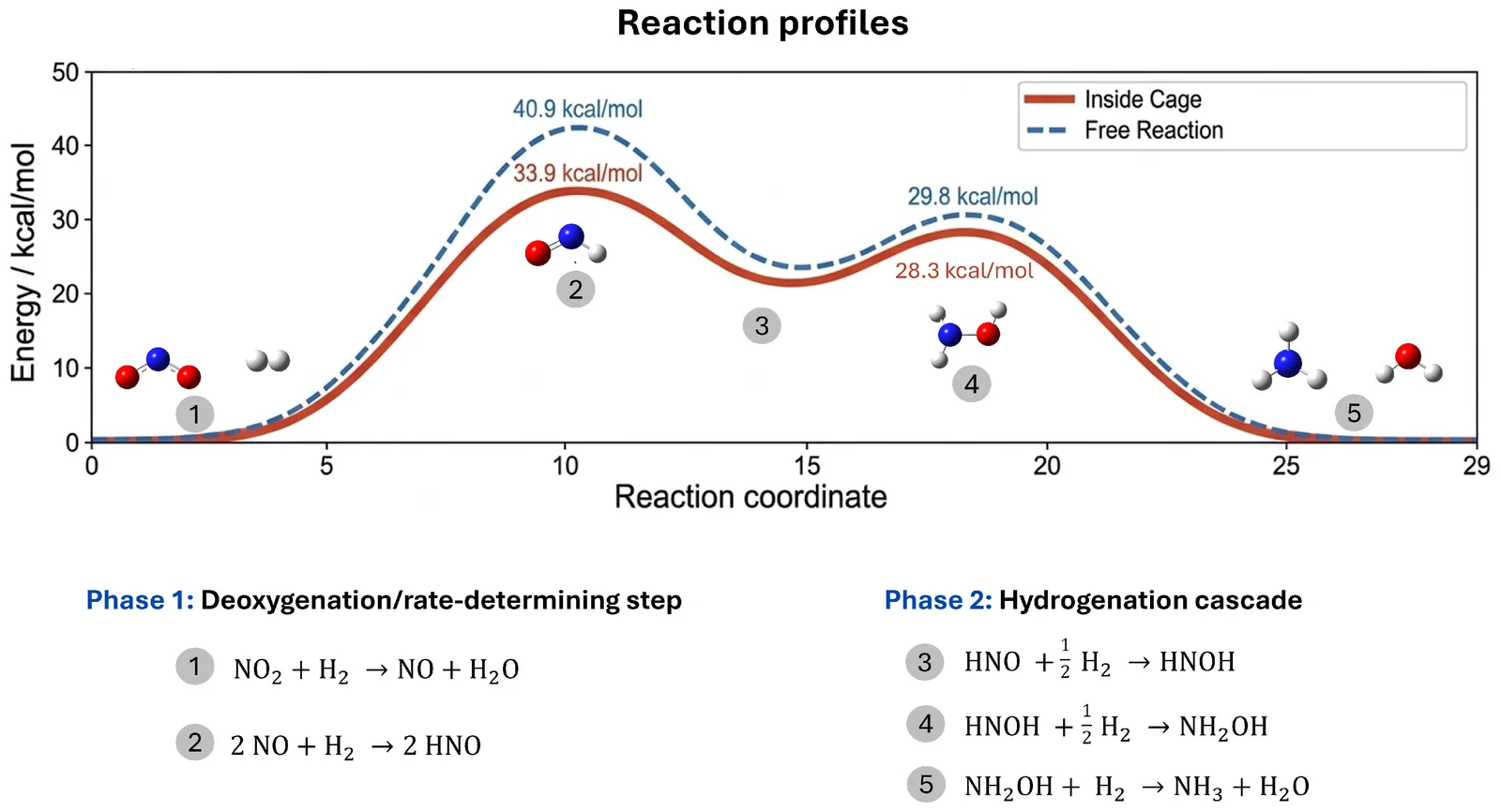
Reaction profiles for the reaction between NO_2_ and H_2_: The dotted line shows the free reaction whereas the red line represents the catalyzed process into the COF cage. In both cases the two-stage process occurs following the mechanism shown above.

To critically evaluate the practical viability of this process, kinetic analysis was performed by mapping the minimum energy pathways. The reaction profiles for the reduction of NO_2_ in the free state and within the M1 cage are presented in Figure 3. Both systems proceed via a two-step mechanism. For the unconstrained free reaction, the rate-determining step exhibits a substantial activation energy (E_a_) of 40.9 kcal/mol. Remarkably, when the reaction is confined within the host cavity (considering the calculated encapsulation energy of 3.9 kcal/mol), the activation barrier is lowered to 33.9 kcal/mol. This calculated value aligns closely with experimentally reported activation barriers for advanced heterogeneous catalytic processes involving localized metal centers in framework environments (42.6 kcal/mol) [34], demonstrating that the framework provides a highly viable kinetic pathway.

A similar kinetic enhancement is observed for the NO reduction pathway, as detailed in Table 2.

The framework lowers the primary activation barrier of NO from 43.7 kcal/mol down to 30.8 kcal/mol. While these catalyzed barriers represent a major energetic reduction compared with the uncatalyzed gas phase, they indicate that the reaction will require moderate thermal activation rather than proceeding spontaneously at ambient room temperature. This quantitative limitation must be noted for practical decentralized reactor designs. The core origin of this catalytic enhancement is the physical confinement effect of the M1 cavity. By pre-organizing individual NO_x_ molecules and isolating them within the rigid cage, the framework enforces spatial separation. This structural isolation suppresses bimolecular coupling pathways that would otherwise form N_2_ or N_2_O side products, effectively funneling the reaction exclusively toward complete hydrogenation (NH_3_).

### 2.4. Multi-Step Mechanism and Charge Transfer Analysis

To avoid optimization bias and accurately map this multi-step mechanism, the reactant complexes were initialized by positioning the H_2_ molecules at a standardized pre-organization distance of 2 Å from the reactive centers of the NO_x_ targets, which represents a verified local minimum on the host–guest potential energy surface.

The reaction starts with the attack of the hydrogen molecule on the NO_2_ species, yielding a NO and water molecules. A hydrogenation reaction occurs on this same NO to provide the intermediate HNO. This is the first step of the reaction. Based on the energy profiles presented in Figure 3, the reaction pathway is grouped into two main kinetic regimes: (1) the deoxygenation/rate-determining step and (2) sequential hydrogenation (Figure 3).

Next, the procedure continues with hydrogenation in two steps, in which a hydroxylamine molecule is formed. Finally, the last hydrogenation reaction on this hydroxylamine species yields the definitive ammonia and water products. These three steps appear in Figure 3.

**Figure 4:**
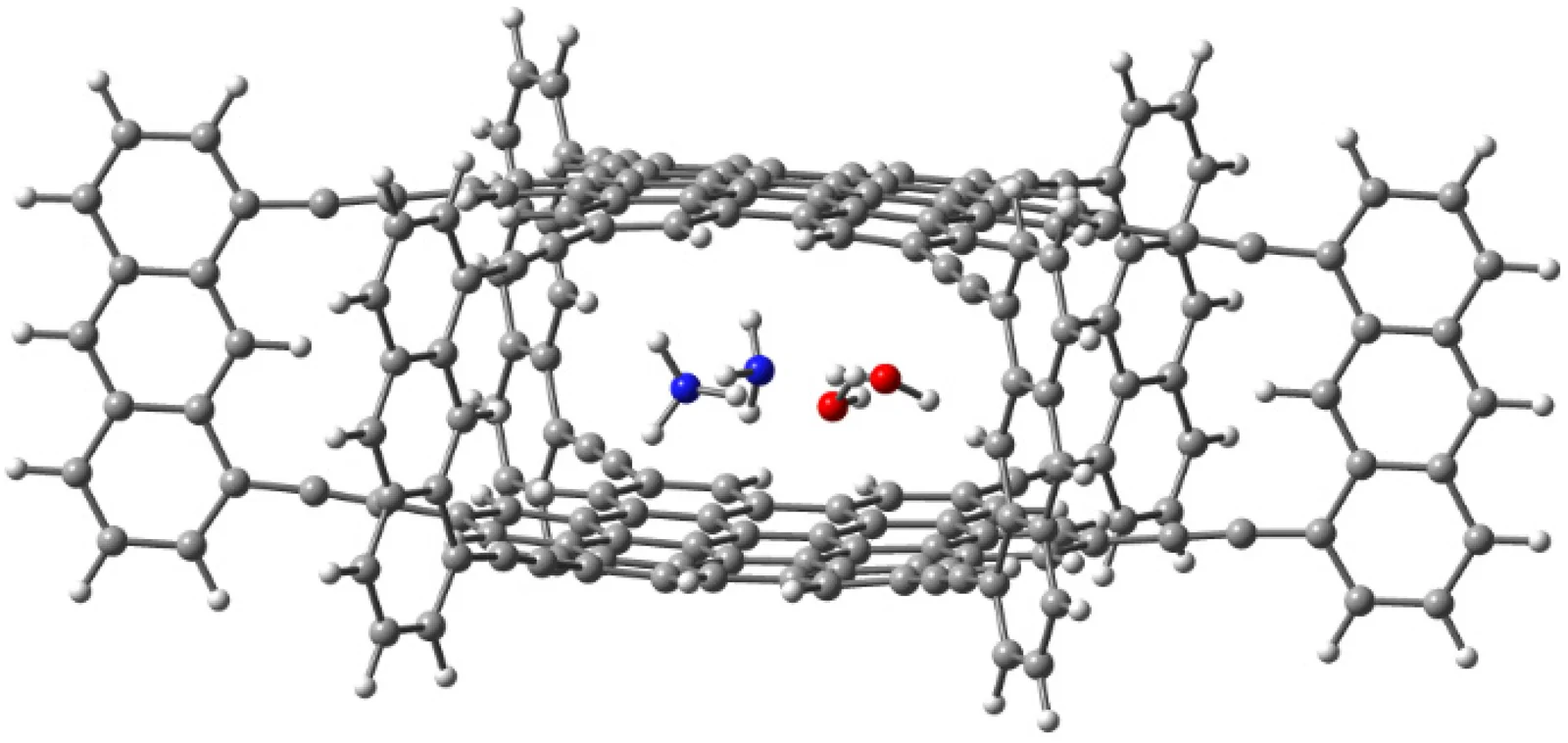
Definitive step of the reaction that produces the COF hollow structure.

The electronic changes driving this process were tracked using Mulliken population analyses as a function of the reaction coordinates. During the formation of the new N–H bonds, the nitrogen atom shifts toward increasingly negative charge states. This synchronized influx of electron density effectively stabilizes the open-shell radical nature of the raw NO_x_ molecules, allowing the system to cross the transition states cleanly and yield stable, closed-shell NH_3_ and H_2_O products into the COF hollow structure (Figure 4).

## 3. Methods

All molecular structures, reaction intermediates, and transition states were geometrically optimized using spin-polarized density functional theory (DFT), as implemented in the Gaussian 16 software package [35]. Electronic exchange and correlation effects were modeled using the MPWB1K hybrid meta-GGA functional theory [36]. This functional theory was specifically selected because of its highly benchmarked accuracy in describing thermochemical kinetics, hydrogen atom transfers, and non-covalent interactions in confined organic geometries [37]. Furthermore, the MPWB1K method has been extensively validated for the treatment of open-shell radical systems where spin properties heavily influence the reactive potential energy surface [19]. The def2SVP basis set was employed for all atomic species. Because the atmospheric pollutants targeted in this work are open-shell radicals, strict spin multiplicity settings were applied to prevent unphysical spin contamination and ensure structural accuracy [38]. Nitrogen monoxide (NO) and nitrogen dioxide (NO_2_) were modeled in their stable doublet ground states (spin multiplicity = 2). Conversely, the hydrogen reducing agent (H_2_) and the host covalent organic framework (COF) cage, designated as M1, were treated as closed-shell singlets (spin multiplicity = 1). Spin conservation was carefully checked along the entire minimum energy pathway (MEP).

To confirm that the optimized structures correspond to true local minima or first-order saddle points on the potential energy surface, vibrational frequency calculations were systematically carried out at the same level of theory. Ground state intermediates were verified by the absence of imaginary frequencies, while transition states were identified by the presence of exactly one imaginary frequency corresponding to the designated reaction coordinate. All reported thermodynamic quantities (including relative electronic energies, enthalpies, and Gibbs free energies) include standard thermal corrections evaluated at T = 298.15 K and P = 1 atm.

The encapsulation energy, i.e., the energy involved in the confinement of NO_x_ and H_2_ into the M1 species, was calculated using the following set of equations:

*E*_ref_ = *E*(M1_free_) + *E*(*NO_x_*) + *nE*(H_2_)

Δ*E*_encapsulation_ = *E*((*NO_x_* − *n*H_2_(M1) − *E*_ref_

All single-NO*_x_* reduction pathways were evaluated on the doublet potential energy surface (2*S* + 1 = 2). For stoichiometric systems involving co-adsorbed 2NO*_x_* species, open-shell singlet (2*S* + 1 = 1) and triplet (2*S* + 1 = 3) states were tested, with the broken-symmetry singlet state identified as the electronic ground state.

## 4. Conclusions

In summary, this study evaluated the catalytic performance of a rigidly constrained, macrocyclic covalent organic framework (M1) for the direct chemical reduction of open-shell NO and NO_2_ pollutants using H_2_ as a reducing agent. Our spin-polarized DFT calculations demonstrate that the physical confinement and high electron density within the eclipsed aromatic cavity fundamentally alter the reaction coordinates. Instead of yielding atmospheric side products or competitive greenhouse gases such as N_2_O, the confined microenvironment drives absolute selectivity toward the synthesis of ammonia (NH_3_) and water (H_2_O), providing a promising “waste-to-value” pathway for green energy carrier production.

Thermodynamic and kinetic analyses reveal that host–guest encapsulation significantly stabilizes the reactive profiles compared with unconstrained gas-phase pathways. The proposed mechanism proceeds via a distinct two-step sequence: (1) the rate-determining step (RDS)—a coordinated hydrogen attack on the terminal oxygen atoms of the encapsulated NO_x_ molecules, leading to N–O bond cleavage and the formation of localized H_2_O; (2) the secondary step—a concerted hydrogenation of the remaining deoxygenated nitrogen centers, which completes the transformation into closed-shell NH_3_ molecules.

The M1 cavity lowers the primary activation barriers from 40.9 kcal/mol to 33.9 kcal/mol for NO_2_, and from 43.7 kcal/mol to 30.8 kcal/mol for NO. NBO and Mulliken charge transfer trends confirm that this kinetic acceleration is mediated by dynamic electron donation from the continuous aromatic framework to the anti-bonding orbitals of the radical guests. While these catalyzed barriers indicate that mild thermal activation is required rather than a spontaneous reaction at ambient room temperature, the exceptional structural integrity and geometric rigidity maintained by the M1 framework throughout the catalytic cycle emphasize its high potential as a recyclable, heterogeneous molecular reactor. This work introduces a clear theoretical roadmap for exploiting host–guest pre-organization in organic cages to synthesize high-capacity clean energy fuels from environmental hazards.

## Figures and Tables

**Table 1:** Gibbs free energy of reaction in kcal mol^−1^.

	STV	FR	IC
NO_2_	−250.9	−248.5	−263.5
NO	−158.6	−156.0	−162.2

**Table 2:** Maximum energies for the NO + H_2_ in kcal mol^−1^.

Configuration	Step 1	Step 2
Free Reaction (FR)	43.7	34.5
Inside Cage (IC)	30.8	25.7

## Data Availability

The authors confirm that the data supporting the findings of this study are available within the article and can be obtained from the corresponding author upon reasonable request.

## Abbreviations

COF	Covalent organic framework
M1	COF molecule proposed as a catalytic chamber
Mt/y	Metric tons per year
MEP	Minimum energy pathway
NICS	Nucleus-independent chemical shift
